# Mapping the impact of the expanded Mexico City Policy for HIV/ family planning service integration in PEPFAR-supported countries: a risk index

**DOI:** 10.1186/s12889-018-6008-2

**Published:** 2018-09-12

**Authors:** Jennifer Sherwood, Alana Sharp, Brian Honermann, Caitlin Horrigan, Meghna Chatterjee, Austin Jones, Chloe Cooney, Greg Millett

**Affiliations:** 1 amfAR The Foundation for AIDS Research, New York City, USA; 2 Planned Parenthood Federation of America, New York City, USA

**Keywords:** HIV, Family planning, Health service integration, Global health policy, The Mexico City Policy

## Abstract

**Background:**

The previously-named Mexico City Policy (MCP) — which prohibited non U.S.-based non-governmental organizations (NGOs) from receiving U.S. family planning (FP) funding if they advocated, provided, counseled, or referred clients for abortions, even with non-U.S. funds — was reinstated and expanded in 2017. For the first time, the expanded MCP (EMCP) applies to HIV funding through the President’s Emergency Plan for AIDS Relief (PEPFAR) in addition to FP funding. Previous, and more limited, iterations of the policy forced clinic closures and decreased contraceptive access, prompting the need to examine where and how the EMCP may impact FP/HIV service integration.

**Methods:**

The likelihood of FP/HIV service de-integration under the EMCP was quantified using a composite risk index for 31 PEPFAR-funded countries. The index combines six standardized indicators from publically available sources organized into three sub-indexes: 1) The importance of PEPFAR for in-country service delivery of HIV and FP services; 2) The susceptibility of implementing partners to the EMCP; and 3) The integration of FP/HIV funds and programming through PEPFAR and USAID.

**Results:**

Countries with the highest overall risk scores included Zambia (3.3) Cambodia (3.2), Uganda (3.1), South Africa (2.9), Haiti (2.8), Lesotho (2.8), Swaziland (2.1), and Burundi (1.5). Zambia’s risk score is driven by sub-index 1, having a high proportion of country HIV expenditures provided by PEPFAR (86.3%). Cambodia and Uganda’s scores are driven sub-index 3, with both countries reporting 100% of PEPFAR supported HIV delivery sites were providing integrated FP services in 2017. South Africa’s risk score is driven by sub-index 2, where roughly 60% of PEPFAR funding is to non U.S.-based NGOs. Of the countries with the highest risk scores, Swaziland, Lesotho, and South Africa, are also in the top quartile of PEPFAR countries for HIV prevalence and unintended pregnancies among young women.

**Conclusion:**

This analysis highlights where and why the EMCP may have the greatest impact on FP/HIV service integration. The possible disruption of service integration in countries with generalized HIV epidemics highlights significant risks. Researchers, national governments, and non-U.S. funders can consider these risk factors to help target their responses to the EMCP and mitigate potential harms of the policy.

## Background

The United States (U.S.) is the largest bilateral funder of international health assistance in the world, contributing 45% of total bilateral funding for family planning (FP), and over a third of all official health development assistance [1]. The majority of U.S. global health funding goes to HIV programs, 80% (~$5.2 billion) of which is provided bilaterally through the President’s Emergency Plan for HIV/AIDS Relief (PEPFAR) [2]. The United States Agency for International Development (USAID) is an important purchaser of commodities, distributing over 630 million condoms and 54 million other contraceptive commodities including oral contraceptives, injectable and intrauterine devices worldwide in 2016 [3].

As a primary funder of FP and HIV activities worldwide, the U.S. policy agenda and funding levels are highly influential in driving global sexual and reproductive health (SRH) activities, including for the integration of FP and HIV service delivery. Recent policy changes under the current U.S. administration have reinstituted and expanded a restriction on U.S. global health assistance, previously called the Mexico City Policy (MCP) [4]. This expanded version of the Mexico City Policy (EMCP), also named *Protecting Life in Global Health Assistance*, restricts non U.S.-based non-governmental organizations (NGO) from receiving U.S. global health assistance unless the organization certifies that it does not provide, counsel, or refer women for abortions – outside of narrow circumstances when the pregnancy arose from rape, incest, or if carrying the pregnancy to term endangers a woman’s life. Non U.S-based NGOs must also certify that they will not promote or advocate for the liberalization of abortion laws, even if using non-U.S. funds for these activities [5]. Unlike other U.S. funding restrictions, which generally dictate allowable activities for that specific funding, this policy dictates what an organization can do as a whole while accepting U.S. funds.

The MCP was last in place from 2001 to 2009, and applied only to non U.S.-based organizations receiving U.S. international family planning/reproductive health (FP/RH) funds – a budget line of about $500 million USD each year. However in the most recent version announced in May 2017, the restriction now applies to nearly all global health assistance – a budget line of $8.8 billion USD in fiscal year (FY) 2017, $5.2 billion of which was appropriated bilaterally for HIV through PEPFAR [4]. This new iteration of the policy (hereafter the expanded Mexico City Policy or EMCP) will take effect over late 2017 and early 2018 as new global health assistance awards are notified and obligated. U.S.-based NGOs and government partners are not required to sign the terms of the EMCP, nor does the policy apply to U.S. contributions to multilaterals including the Global Fund to fight  AIDS Tuberculosis, and Malaria (Global Fund) and the United Nations. However, U.S.-based NGOs must enforce the funding restrictions of the EMCP with its non U.S.-based NGO sub-partners. Signing the provisions of the EMCP is not voluntary: those non-U.S.-based NGOs and private universities that do not sign will lose U.S. global health assistance funding.

Under previous iterations of the policy, major reproductive health providers, such as International Planned Parenthood Federation, lost funding due to irreconcilable conflicts with the policy regarding service provision and ethics – which had national implications for SRH service provision. In Ghana, Planned Parenthood Association of Ghana (PPAG) was responsible for 21% of facility-based FP services in 2001. After the loss of U.S. funding, which reduced its budget by 40%, 57% of PPAG clinics were closed – resulting in a 10% reduction in national contraceptive availability [6]. Decreased access to contraception, especially in rural areas, resulted in increased unintended pregnancy rates [6]. A multi-country analysis utilizing Demographic Health Survey (DHS) data showed that in countries with higher exposure to the MCP, abortion rates increased compared to countries with low exposure [7]. The authors posit this is due to a reduction in contraception supply, resulting in an increase in unintended pregnancies. Qualitative evidence from multiple settings support these findings, recording clinic closures, staff and resource cut backs, and termination of shipments of contraceptive commodities as a result of the MCP [8, 9].

Given the expansive nature of the EMCP, which implicates substantially more implementing partners than previous years, the likelihood of funding cuts to key SRH organizations that cannot comply with the policy’s provisions is increased. Cuts to PEPFAR partners that are providing integrated FP and HIV services could have major public health implications, especially for women living with HIV (WLHIV). FP service provision is a key public health intervention that has had a documented impact on the prevention of perinatal transmission of HIV, in addition to preventing unintended pregnancies, reducing the number of abortions overall and the number of unsafe abortions, contributing to birth spacing, and lowering infant and maternal mortality rates [10–12]. WLHIV have especially high levels of unmet need for FP, with multiple studies in sub-Saharan Africa estimating that around 60% of pregnancies to WLHIV are unintended [13–15]. The integration of FP into HIV service sites is a key way to reach WLHIV and has been shown to increase the use of modern contraception among WLHIV in several sub-Saharan African settings [16–18]. By prompting clinic closures and funding cuts to key SRH providers, the EMCP has the potential to increase unmet need for FP for women, including those living with HIV, thereby increasing unintended pregnancies, the number of infants born with HIV, and other associated health concerns.

PEPFAR-supported HIV service delivery sites are a vital platform to reach women and couples living with or at risk of HIV, and provide additional FP services to meet their SRH needs. USAID FP funding can be used to co-fund PEPFAR service delivery mechanisms, generally being used to support FP provision or purchase contraceptive commodities at PEPFAR-supported sites [19]. In recent years, PEPFAR has increasingly prioritized the bi-directional integration of HIV and FP services as an effective and cost-effective way to prevent unintended pregnancies for WLHIV and increase HIV testing and linkage to care at family planning sites. PEPFAR guidance from 2009 onward acknowledges the importance of preventing unintended pregnancies in lowering the rate of new HIV infections, and endorses the use of PEPFAR funds for family planning counseling and referrals for contraceptives as part of programing for preventing mother to child transmission (PMTCT) [20]. PEPFAR, in collaboration with private sector partners, also launched DREAMS – a new initiative to prevent HIV in adolescent girls and young women and key to their approach is an integrated, evidence-based package of interventions [21]. These shifts are promising indications that PEPFAR is organizationally moving towards more integrated service delivery. However, FP/HIV integrated sites are not yet universal, and questions remain as to the impact of the EMCP and changes to overall U.S. global health funding on service integration.

Given the unprecedented expansion of the MCP to HIV funding, as well as previous evidence that the policy decreased access to FP, there is a need to examine where and how the EMCP may impact FP/HIV service integration in order to appropriately monitor health impacts of the policy and direct efforts to mitigate harm. This paper proposes an index to measure country risk to de-integration of FP/HIV services under the EMCP using available national and PEPFAR-specific data. To our knowledge this is the first index to attempt to measure where, and for what reasons, the EMCP will be most impactful to FP/HIV service integration.

## Methods

### Country selection

All countries that received PEPFAR funds in 2017 were included in this analysis (*n* = 31). Due to difficulties in obtaining disaggregated data at the country level, regional PEPFAR programs were not included.

### Development of risk index for FP/HIV integration due to the EMCP

Country composite scores were built to measure the likelihood of disruptive impact to FP/HIV integration, or risk to integration, under the implementation of EMCP. All indicators were standardized using z-scores, allowing for cross-indicator comparisons and for the creation of a composite index built from several different indicator scales. A separate mean and standard deviation are calculated for each indicator (*x*), and a separate z-score is calculated for each country-indicator (*x*_*i*_). The z-scores are calculated as follows:

$$ {z}_i=\frac{x_i-\overline{x}}{\sigma_x} $$

Risk scores on this standardized scale therefore represent relative risk in relation to other countries as opposed to a measure of absolute risk. As such, “0” represents average risk within this group, while positive and negative scores represent above or below average risk respectively, as opposed to complete or no risk of de-integration. This risk index combines three major dimensions hypothesized to increase a country’s vulnerability to de-integration of FP/HIV services under the EMPC: 1) The importance of PEPFAR for in-country service delivery of HIV and FP services, 2) The susceptibility of implementing partners (IPs) to the EMCP, and 3) The integration of FP/HIV funds and programming. Under this framework, countries that are more reliant on U.S. global health funding are hypothesized to be more affected by U.S. funding restrictions, as represented through sub-index 1. Additionally, it is hypothesized that in countries where a greater proportion of PEPFAR partners are required to sign the provisions of EMCP, and where those provisions are at odds with more liberal national abortion laws, more disruption to IPs is expected. This is represented by sub-index 2. Lastly, current levels of FP integration in PEPFAR-supported HIV service sites represent a higher risk of de-integration if U.S. funding streams are disrupted, which is represented in sub-index 3. Each of these three sub-indices is measured through two indicators, which are converted into z-scores and summed to give one standardized score for each sub-index. The overall risk score for the country is then the sum of the z-scores for the three sub-indices:

$$ {\displaystyle \begin{array}{l} Total\ score: FP/ HIV\  Integration\ Risk\ Index=\\ {}\left[\mathrm{Importance}\ \mathrm{of}\ \mathrm{PEPFAR}\ \mathrm{for}\ \mathrm{service}\ \mathrm{delivery}\right]+\\ {}\left[\mathrm{Susceptibility}\ \mathrm{of}\ \mathrm{IPs}\ \mathrm{to}\ \mathrm{the}\ \mathrm{EMCP}\right]+\\ {}\left[\mathrm{Integration}\ \mathrm{of}\ \mathrm{U}.\mathrm{S}.\mathrm{FP}/\mathrm{HIV}\ \mathrm{funds}\ \mathrm{and}\ \mathrm{programming}\right]\end{array}} $$

### Indicators for sub-index 1: The importance of PEPFAR for in-country service delivery of HIV and FP services

PEPFAR is an important provider of HIV services in every investment country. However, the proportion of total services that are delivered by PEPFAR versus other funders like the Global Fund or national governments varies widely by country. In contexts where PEPFAR is a primary provider of HIV services, changes to PEPFAR contracting requirements are more likely to be disruptive to IPs and service delivery.

The U.S. contribution to national HIV and FP services is approximated by an indicator for the proportion of total national HIV expenditures that are provided by PEPFAR in 2017. This indicator is reported by each country in the 2017 PEPFAR Strategic Direction Summaries (SDS) [19]. Higher proportions of HIV expenditures supported by PEPFAR indicates a greater comparative role in HIV service delivery. Secondly, this sub-index includes an indicator for the amount of FP/RH funding from USAID and PEPFAR and per-capita, calculated as the total funding from the two funding streams divided by the total country population. All funding data are from FY2017, representing planned bilateral HIV funding through PEPFAR and planned FP/RH funds by USAID [22, 23]. Total country population is taken from the 2017 global estimates from World Bank [24]. This measure approximates the magnitude of in-country U.S-supported program, with larger programs reaching/affecting more IPs. Higher scores in this sub-index indicate a higher proportion of PEPFAR provided services and funding, and therefore increased risk to service disruption due to changes in PEPFAR contracting requirements.

### Indicators for sub-index 2: Susceptibility of implementing partners to the EMCP

The EMCP specifically applies to non U.S.-based IPs. In countries where a plurality of PEPFAR grants are to non U.S.-based NGOs, as opposed to U.S.-based organizations, there is a higher likelihood of the policy producing impact. Additionally, in contexts where local law allows for abortion services outside of the limited circumstances permitted under the EMCP, there is greater likelihood of local partner non-signature – as agreeing to the policy would mean restricting available services beyond what is allowable under local law. For example, in a country where abortion is legal and is a standard part of available SRH services, compliance with the EMCP would mean more radical departures from normal operations and increase the likelihood of partner non-signature. This sub-index includes two indicators to measure approximate susceptibility of IPs to changes in U.S. policy: 1) The proportion of PEPFAR funding contracted to non-U.S. partners (through primary or sub-contracts), and 2) A quantitative score for abortion legality, ranging from 0.0 (only legal in cases that are also allowed under the EMCP) to 1.0 (available upon request).

The total estimated funding to non U.S-based IPs combines the known funding allocated to non U.S.-based prime partners from 2017 as well as the estimated proportion sub-contracted to non U.S-based sub-partners based on historical rates. For prime partners, the proportion of PEPFAR funding to non U.S.-based organizations was calculated with data from PEPFAR 2017 country operational plans (COPs) [22] by summing the total funds allocated directly to non U.S.-based NGOs, faith based organizations (FBOs), private contractors, and universities through primary grant agreements. Sub-partner allocation amounts are not available after 2009; however, these are estimated by back-calculating the average percentage of each funding mechanism that was retained by the prime partner (retention rate) in 2007–2009 and subtracting this from 100% to estimate the amount sub-granted. The total estimated amount which is sub-granted by non U.S.-based primes was then multiplied by the percent of sub-partner funding that goes to non U.S.-based sub-partners (local sub-partner split) to estimate the amount sub-granted to non U.S-based sub-partners and applied to the total U.S. partner total funding as follows:

$$ {\displaystyle \begin{array}{l} Total\ to\ non\ U.S.- based\  IPs=\\ {} Funding\ to\ non\ U.S.- based\ partners\ through\ primary\ grant\ agreements+\\ {}U.S. partner\ {total\ funding}^{\ast }\ {\left(1- retention\ rate\right)}^{\ast }\ \left( local\  sub- partner\ split\right)\end{array}} $$

Data on abortion legality by country are taken from the Center for Reproductive Rights, as recently summarized by the Kaiser Family Foundation [25]. To measure the variation in abortion legality, countries with abortion laws which allow abortion in the same or fewer circumstances than in the EMCP were given a score of zero (countries that allow abortion only in cases of rape, incest, and/or if carrying the pregnancy to term threatens the life of the woman). For each circumstance of increasing abortion legality beyond the EMPC a country was given + 0.2 points. For example, in Zimbabwe abortion is legal to save a woman’s life, in cases of rape or incest, to preserve a women’s physical health, or to preserve her mental health; and was given a score of 0.4 to indicate two circumstances in which abortion is legal beyond the EMCP. Countries with fully legal abortion (available on request) were given a score of 1.0. Conceptually, increasingly higher scores (0.2 to 1.0) will indicate more legal abortion and increased disparity with abortion restrictions under the EMPC. Standardized scores for both indicators were summed to create a sub-index score and contribute to the full risk index, with higher sub-index scores estimating more susceptibility of IPs to the EMCP.

### Indicators for sub-index 3: Integration of U.S. FP/HIV funds and programming

Of primary interest in this analysis are indicators for FP/HIV integration within PEPFAR programming. Countries with higher levels of FP and HIV service integration are those where de-integration is most likely to occur if current FP referral systems or services are disrupted due to non-signature under the EMCP. Given this, higher proportions of FP/HIV integrated sites and programming, indicated by higher scores in this sub-index, indicate greater risk for service de-integration or disruption. FP/HIV integration in PEPFAR programming was measured through: 1) The proportion of total PEPFAR mechanisms which are co-funded with USAID FP funds in 2017, and 2) The proportion of PEPFAR supported clinics that report FP/HIV integrated service delivery in 2017.

Data on PEPFAR/USAID co-financing were extracted from PEPFAR SDS, and are only available in countries where USAID is investing in FP services (13/31 countries). Indicator scores are calculated as the number of PEPFAR mechanisms which report co-financing with USAID funding divided by the total number of mechanisms. Although there is variation between countries, these co-funded mechanisms’ primary objectives are to support/increase FP service delivery, with some indicating commodity purchasing of condoms and essential medicines [26]. Since PEPFAR funds are not used to purchase contraceptive commodities other than condoms, co-financing with USAID FP funds is an important indicator of contraceptive availability at PEPFAR-supported HIV sites. Data on the proportion of PEPFAR-supported HIV service delivery sites, which provide integrated FP services, were extracted from PEPFAR program monitoring data synthesized through the amfAR COP database [22]. A service delivery point is counted as providing integrated voluntary family planning services if the site: 1) Assesses family planning needs through routine screening, 2) Provides voluntary family planning counseling, and 3) Provides contraception on-site or detailed referral for a broad range of modern contraception methods [27]. The indicator is calculated as the percent of the total number of PEPFAR-supported HIV service delivery points for PMTCT, care, and treatment that are providing integrated FP services under this definition.

## Results

### Risk index

Of the 31 PEPFAR countries included in the index, Zambia had the highest score for risk of de-integration (score = 3.3). This score was driven primarily by a high score in the sub-index 1: Importance of PEPFAR for service delivery. Cambodia (score = 3.2) and Uganda (score = 3.1), ranked second and third on the risk index, and were primarily vulnerable to the EMCP due to high scores on sub-index 3: Integration of FP and HIV funds and programming. South Africa ranked fourth overall (score = 2.9), with primary risk for de-integration driven by sub-index 2: Susceptibility of IPs to the EMCP. The majority of countries with overall risk scores in the top quartile are located in Africa and include: Zambia, Uganda, South Africa, Lesotho, Swaziland and Burundi. Non-African countries with high risk scores include Cambodia and Haiti (Table 1).

**Table 1:** Full FP/HIV Integration Risk Index for PEPFAR funded countries in 2017

		Importance of PEPFAR for service delivery sub-index			Susceptibility of Implementing Partners to EMCP sub-index			Integration of U.S FP/HIV funds and programming sub-index		
Country	Full Index Score	Index 1 z-score	% from PEPFAR^a^	Per-capita U.S. funding^b^	Index 2 z-score	% to non-U.S. NGO^c^	Abortion legality^d^	Index 3 z-score	% Co-funded mechanism^e^	% FP/HIV sites^f^
Zambia	3.3*	3.1*	86.3	18.5	0.1	21.8	1,3,5,6	0.1	6.1	83.4
Cambodia	3.2*	− 1.1	29.3	1.0	1.0*	18.9	1,2,3,4,5,6,7	3.2*	26.7	100
Uganda	3.1*	0.9	62.0	6.5	−0.2	43.5	1	2.5*	20.3	100
South Africa	2.9*	− 0.7	21.9	8.0	3.5*	60.6	1,2,3,4,5,6,7	0.1	0.0	99.9
Haiti	2.8*	2.0*	81.3	8.7	− 1.1	28.8	1	1.9*	15.8	98.9
Lesotho	2.8*	2.0*	39.5	28.3	0.6*	48.9	1,2,5	0.1	0.0	100
Swaziland	2.1*	2.7*	16.2	45.4	− 0.3	15.0	1,2,3,4,5	−0.3	0.0	90.5
Burundi	1.5*	− 0.6	40.0	1.3	0.4	44.8	1,3	1.7*	29.4	58.5
Malawi	1.3	0.0	45.4	4.7	0.3	53.2	1	1.0*	10.5	91.6
Kenya	1.3	1.2	58.4	10.6	−0.8	24.6	1,3	0.9*	9.4	94.3
Mozambique	0.9	1.9*	74.0	11.6	1.0*	36.7	1,2,3,4,5	− 2.1	4.0	41.5
Tanzania	0.7	1.2*	67.0	7.2	− 1.5	21.7	1	1.1*	14.8	83.0
Zimbabwe	0.1	−0.8	21.1	7.8	0.9*	44.9	1,2,3,4	− 0.1	2.9	88.7
Ethiopia	−0.2	0.3	52.0	4.4	−1.4	5.7	1,2,3,5	0.9*	9.5	93.3
Rwanda	−0.9	0.0	43.0	5.5	−1.6	10.3	1,2,3	0.8	5.9	99.6
Côte d’Ivoire	−1.0	1.4*	77.0	4.7	−0.7	35.3	1	−1.7	0.0	60.5
Democratic Republic of the Congo	−1.9	−0.4	44.3	1.1	−0.5	39.5	1	−1.0	16.7	31.5
Nigeria	−2.6	0.7	64.0	3.3	−1.8	16.8	1	−1.5	0.0	65.2
India	−3.1	−2.1	7.0	0.2	2.7*	56.2	1,2,3,4,5,6	−3.7	6.3	0
Namibia	−3.3	1.3*	27.0	26.6	− 0.3	15.0	1,2,3,4,5	−4.4	0.0	0
South Sudan	−3.4	−0.3	45.0	1.7	−0.9	31.7	1	−2.1	0.0	50.0
Vietnam	−3.8	−0.8	36.0	0.3	1.4*	25.6	1,2,3,4,5,6,7	−4.4	0.0	0
Ghana	−4.0	−1.6	17.8	0.7	0.4	26.2	1,2,3,4,5	−2.7	14.3	0
Botswana	−4.3	0.5	28.4	17.4	−0.3	13.9	1,2,3,4,5	−4.4	0.0	0
Indonesia	−4.7	−2.0	9.0	0.3	1.7*	67.9	1,2,5	− 4.4	0.0	0
Papua New Guinea	−5.8	−0.9	33.0	0.7	−0.5	39.1	1	−4.4	0.0	0
Ukraine	−6.0	−1.8	13.6	0.8	0.2	4.2	1,2,3,4,5,6,7	−4.4	0.0	0
Angola	−6.2	−1.5	20.9	0.6	−0.4	41.4	1	−4.4	0.0	0
Dominican republic	−6.3	−1.2	27.0	0.7	−0.7	36.2	1	−4.4	0.0	0
Cameroon	−7.6	−1.6	15.5	1.7	−1.6	11.4	1,2,3	−4.4	0.0	0
Burma	−8.7	−1.9	12.0	0.2	−2.4	6.0	1	−4.4	0.0	0
Cross – Country Average			39.2	7.4		30.5	n/a		6.6	49.4

*Top quartile in sub-index

a: Percent of country HIV expenditures funded by PEPFAR

b: Total PEPFAR and USAID FP/RH funding per total country population ($USD)

c: Percent of PEPFAR funds allocated to non U.S.-based NGOs, FBOs, and private contractors as prime or sub-grantees

d: Abortion legal in following circumstance: 1. To save a woman’s life, 2. In the case of rape or incest, 3. To preserve a women’s physical health, 4. To preserve a women’s mental health, 5. Due to fetal impairment, 6. For economic or social reasons, 7. On request [25]

e: Percent of total PEPFAR mechanism which are co-funded with USAID FP/RH funds

f: Percent of total PEPFAR-supported HIV service delivery points for PMTCT, care and treatment providing integrated family planning services

Sub-index 1 signifies countries with relatively high levels of PEPFAR funding and HIV services provided by the U.S. government. In Zambia, 86.3% of HIV expenditures are provided by PEPFAR, followed by Haiti at 81.3%. Swaziland and Lesotho among the countries with the highest per-capita U.S HIV and FP funding. With the exception of Haiti, all of the countries with scores in the top quartile of sub-index 1 are located in sub-Saharan Africa: Zambia, Swaziland, Lesotho, Haiti, Mozambique, Cote d’Ivoire, Namibia, and Tanzania.

Countries with top quartile scores in sub-index 2 include: South Africa, India, Indonesia, Vietnam, Cambodia, Mozambique, Zimbabwe, and Lesotho. South Africa leads this sub-index with a score of 3.5, having both legal abortion upon request and a large fraction of PEPFAR funding contracted to non U.S.-based organizations (60.6%). India, where over 56% of PEPFAR funds go to non U.S.-based partners, ranks second on this sub-index. All countries with legal abortion on request ranked in the top quartile of sub-index 2 except for Ukraine, where only 4.2% of PEPFAR funds go to non U.S.-based NGOs.

Sub-index 3 describes where U.S. government supported FP and HIV funds and programming are most integrated. Full index risk scores for Cambodia, Uganda, Haiti, and Burundi are driven by high scores to sub-index 3, with all countries reporting high proportions of FP/HIV integrated sites (58% in Burundi – 100% in Cambodia and Uganda) and co-funded PEPFAR/ USAID mechanisms (15% in Haiti – 29% in Burundi). Countries with high scores in sub-Index 3 include: Cambodia, Uganda, Haiti, Burundi, Tanzania, Malawi, Kenya, and Ethiopia. All countries that scored in the top quartile received USAID FP funding and reported that at least 50% of PEPFAR-supported sites provided comprehensive FP services or referrals.

### Figures: Percent of unintended pregnancies and HIV prevalence vs. integration risk index score

**Fig. 1:**
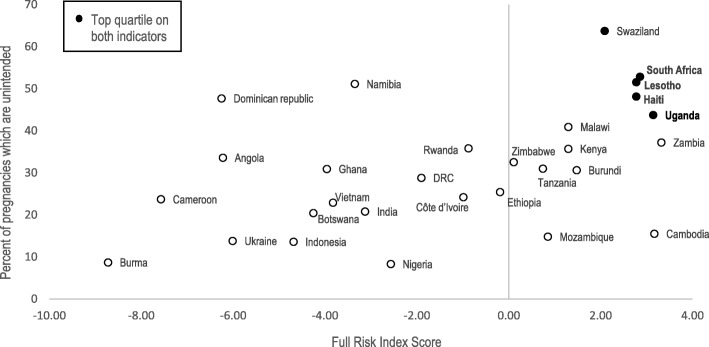
Percent of Unintended Pregnancies vs. Full Integration Risk Index Score

**Fig. 2:**
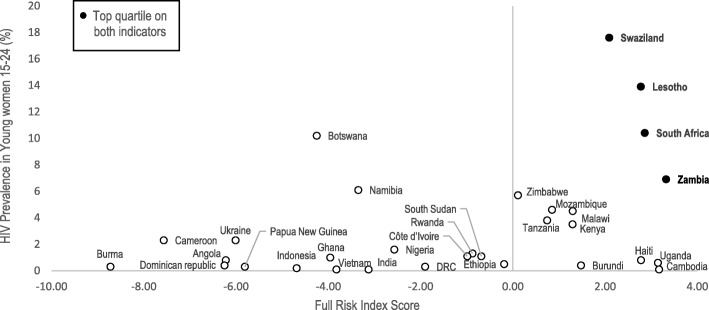
HIV Prevalence in Young Women (2017) vs. Full Integration Risk Index Score

Full risk index scores were plotted against the estimated proportion of pregnancies which are unintended (reported as not wanted or wanted later) and estimated HIV prevalence in young women and girls aged 15–24 in 2017 [28, 29]. Countries with both risk index scores and HIV prevalence or unintended pregnancy rates in the top quartile are highlighted (Figs. 1 and 2).

The proportion of pregnancies that are unintended ranged from 8.3% in Nigeria (2013) to 63.7% in Swaziland (2007). No DHS data on these indicators were available for Papua New Guinea or South Sudan. Of countries with top quartile proportions of unintended pregnancies (> 42%) five countries also ranked in the top quartile on the FP/HIV risk index; Swaziland, South Africa, Lesotho, Haiti, and Uganda. Countries with top quartile HIV prevalence (> 4.6%), and top quartile risk index scores included; Swaziland, Lesotho, South Africa, Zambia.

## Discussion

Among the countries with the highest overall risk to FP/HIV integration, Swaziland, Lesotho, and South Africa consistently ranked in the top quartiles on comparative risk indicators for HIV prevalence and unintended pregnancy rates (Figs. 1 and 2). In Swaziland, where over 63% of pregnancies are unintended and HIV prevalence among young women is 17.6%, increases to unintended pregnancy rates for WLHIV would have severe health consequences. Unintended pregnancies increase women’s risk of maternal mortality, especially for WLHIV who are at an estimated eight times greater risk for pregnancy-related death than HIV-negative women [30]. Unintended pregnancies to WLHIV are also a primary contributor to new infant HIV cases, with previous models demonstrating that 90,000 infant HIV infections could be averted globally each year by preventing unintended pregnancies [31]. Although rises in unintended pregnancy rates would be damaging in all countries, those with high rates of unintended pregnancy, HIV prevalence, and projected risk of de-integration of FP/HIV services are a natural priority for harm reduction efforts and monitoring of health outcomes.

South Africa ranked among the top countries for overall risk to FP/HIV integration due to a high proportion of PEPFAR funding flowing to non U.S.-based NGOs and having liberal abortion laws. Although having abortion legally permissible in more circumstances than those permitted under the EMCP does not necessarily predict where non-signature of the EMCP will occur, it does indicate where local law and the provisions of the EMCP are most disparate. Local partners that offer abortion services, counseling, or referrals in accordance with local law may be less willing to restrict services based on changing U.S. contract provisions. South Africa has both high HIV prevalence among young women (10.4%) and over 50% of pregnancies reported as unintended. In large countries like South Africa, even marginal increases in unintended pregnancy rates among WLHIV could have major national and regional consequences for maternal and child health and mother-to-child HIV transmission.

Sub-index 3 highlights countries who have the most evidence of FP/HIV integration, including higher proportions of PEPFAR sites offering FP services and of PEPFAR/ USAID co-funded mechanisms. Since PEPFAR does not purchase contraceptive commodities other than condoms, co-financing of PEPFAR mechanisms with USAID FP funds is vital to support the full integration of FP/HIV services and is an indicator of contraceptive availability at PEPFAR-supported sites. Ten of the 31 countries in this index report over 90% of HIV sites to be integrated with FP services. If these sites no longer offer FP commodities or referrals, due to cutbacks of USAID funding, lack of appropriate FP referral sites, or simply misinterpretation of the policy, many WLHIV could lose access to FP commodities, counseling or information. While signing the EMCP does not explicitly restrict the provision or referral of non-abortion FP services, systems may be disrupted as funds are diverted away from organizations who were historically responsible for providing FP services, and receiving referrals. In Haiti and Uganda, over 40% of pregnancies are unintended; if contraceptive commodities become less available through USAID or referral systems for FP are disrupted under the EMCP, unintended pregnancy rates could rise further, with serious maternal and child health implications.

Although quantitative data are limited, previous research on the historical implementation of the MCP shows that the policy is linked to decreased access to contraception, leading to increased unintended pregnancy and abortions [6, 7]. The effect was especially pronounced for rural woman, whose healthcare options were already more limited [6]. Country case studies have shown that the MCP diverted funds away from major FP providers, resulting in budget cuts, clinic closures, and reduced scope of clinical and outreach activities [8]. Under the previous iteration of the policy (2001–2009), USAID-provided shipments of condoms to Lesotho were ceased after Planned Parenthood Lesotho could no longer receive U.S. funds in 2001, and another suitable distribution partner could not be found [8]. This risk index highlights Lesotho as a country at high risk for de-integration of FP/HIV services under the EMCP. Reductions in condom availability in areas like Lesotho, with HIV prevalence of 25%, is a public health emergency.

This risk index allows for cross-country comparisons to examine where and why the EMCP may have the greatest implications for FP/HIV integrated service delivery. Unlike previous analyses of MCP, which focused strictly on access to FP services, this index measures the impact of the EMCP on the integrated provision of FP/HIV services. National access to FP services will depend on several other factors not measured here, including pre-existing unmet need for contraception and the overall proportion of FP services provided by the U.S. government. However, de-integrating FP and HIV services can lead to decreasing access to FP services, especially for WLHIV who are more likely to use modern contraception while accessing FP/HIV integrated care [17, 18].

Identifying where non-signing partners are operating and their relative responsibility for service provision is an important area for future work and would add precision to the index. Additionally, building on this index to track U.S. resource flow and health outcomes in areas with high susceptibility could inform non-U.S. resource distribution to mitigate potential harms of the policy and inform future debates about whether this policy is congruent with U.S. global health goals. In recognition that the service gaps created by the EMCP may extend well beyond the provision of safe abortion services, other non-U.S. donor governments that have traditionally supported FP, including the United Kingdom, the Netherlands, Sweden, Canada, and recipient country governments, can increase their support of comprehensive SRH programming including FP/HIV integrated services to mitigate potential policy-related harms.

### Limitations

The development of this index has a few major limitations. Firstly, it does not attempt to predict which service providers will not sign the EMCP or quantify their relative contribution to service delivery. While the index does track overall susceptibility of IPs to the policy, without specific data on non-signing partners it is uncertain where partner disruption, and therefore service disruption, will be the highest. Similarly, the index does not include data on non-U.S. FP funding, the availability of which will play a key role in an organization’s decision to take U.S. funds. Additionally, all indicators in the index are equally weighted, with the total risk index score being a simple sum of all indicators. However, the true contribution of each indicator is unknown and so attempts to add weights to the indicators based on estimation would not guarantee a more accurate risk index score.

While every attempt was made to report the most standardized variables possible, some country documents varied in the ways that indicators were reported. For example, some SDSs reported the total percentage of HIV expenditures provided by PEPAR and some reported only raw dollar amounts, such that the proportional calculation had to be done manually. In these circumstances, it is not clear if there were other country HIV funding that would have contributed to the denominator, but was not available in the SDS table. Finally, data on PEPFAR sub-partners are limited in recent years by their removal from PEPFAR’s COPs in 2010 and are thus historical data.

## Conclusion

This index points to higher risk for the de-integration of FP and HIV services in several regions due to the EMCP. Researchers, national governments, and non-U.S. funders can consider these risk factors to help target their responses to the EMCP and mitigate potential harms of the policy. To the extent possible, organizations should continue to refer, counsel, and provide FP as part of a comprehensive strategy to meet population SRH needs. PEPFAR teams should continue to set targets for FP integration, and countries where targets are not available should initiate tracking FP integration as a valuable health indicator. In areas where restrictions on U.S. funds impact the availability of FP service providers or availability of commodities, these effects must be documented.

Meeting the contraceptive needs of women, including those living with HIV, is imperative for preventing unintended pregnancies, averting new infant HIV cases, spacing desired births, and improving health outcomes for women and children. HIV service sites are a natural venue to reach women at risk or living with HIV, making the integration of FP/HIV at U.S.-supported service sites a priority to prevent unintended pregnancies for this population. Data on the effects of the EMCP on FP/HIV integration and related health outcomes must be collected annually as long as the policy is in place and considered in the development of future U.S. global health policy.

## Abbreviations

COP: Country Operational Plan
DHS: Demographic Health Survey
DREAMS: Determined Resilience Empowered, AIDS-free, Mentored Safe
EMCP: Expanded Mexico City Policy
FBO: Faith Based Organization
FP: Family Planning
FY: Fiscal Year
HIV: Human Immunodeficiency Virus
IP: Implementing Partners
MCP: Mexico City Policy
MTCT: Maternal to Child Transmission
NGO: Non-governmental Organization
PEPFAR: President’s Emergency Plan for AIDS Relief
PPAG: Planned Parenthood Association of Ghana
SDS: Strategic Direction Summaries
SRH: Sexual and Reproductive Health
USAID: United States Agency for International Development
WLHIV: Women living with HIV
